# Delayed *Plasmodium falciparum* clearance following artesunate-mefloquine combination therapy in Thailand, 1997–2007

**DOI:** 10.1186/1475-2875-11-296

**Published:** 2012-08-28

**Authors:** Saowanit Vijaykadga, Alisa P Alker, Wichai Satimai, John R MacArthur, Steven R Meshnick, Chansuda Wongsrichanalai

**Affiliations:** 1 Bureau of Vector Borne Diseases, Department of Diseases Control, Ministry of Public Health, Nonthaburi, Thailand; 2 Division of Infectious Diseases, Department of Medicine, University of North Carolina, Chapel Hill, NC, USA; 3 US Agency for International Development Regional Development Mission for Asia (USAID/RDMA), Bangkok, Thailand; 4 Malaria Branch, Centers for Disease Control and Prevention, Atlanta, Georgia, USA; 5 Department of Epidemiology, Gilings School of Public Health, University of North Carolina, Chapel Hill, NC, USA

**Keywords:** Malaria, drug resistance, parasite clearance

## Abstract

**Background:**

There is concern that artesunate resistance is developing in Southeast Asia. The purpose of this study is to investigate the prevalence of parasitaemia in the few days following treatment with artesunate-mefloquine (AM), which is an indirect measure of decreased artesunate susceptibility.

**Methods:**

This is a retrospective analysis of 31 therapeutic efficacy studies involving 1,327 patients treated with AM conducted by the Thai National Malaria Control Programme from 1997–2007.

**Results:**

The prevalence of patients with parasitaemia on day 2 was higher in the east compared to the west (east: 20%, west: 9%, OR 2.47, 95% CI: 1.77, 3.45). In addition, the prevalence of day-2 parasitaemia increased over time (OR for each year = 1.10, 95% CI: 1.03, 1.19). After controlling for initial parasitaemia and age, year and region remained important determinants of day-2 parasitaemia (OR for region = 3.98, 95%CI 2.63, 6.00; OR for year = 1.28, 95%CI: 1.17, 1.39). The presence of parasitaemia on day 2 and day 3 were specific, but not sensitive predictors of treatment failure.

**Discussion:**

Delayed resolution of parasitaemia after AM treatment increased in eastern Thailand between 1997 and 2007, which may be an early manifestation of decreased artesunate susceptibility. However, clinical and parasitological treatment failure after 28 days (which is related to both mefloquine and artesunate decreased susceptibility) is not changing over time. The presence of parasitaemia on day 2 is a poor indicator of AM 28-day treatment failure.

## Background

Artemisinin combination therapy (ACT) is the first-line treatment for uncomplicated falciparum malaria in most of the world [1]. As a result, the emergence of artemisinin resistance would have worldwide implications. Southeast Asia has been the focus of monitoring for artemisinin resistance, as these compounds were first used as monotherapy in Vietnam over two decades ago and later in Thailand and Cambodia in combination with mefloquine [2-4]. In addition, the Thai-Cambodian border has a long history of anti-malarial resistance, as chloroquine, sulphadoxine-pyrimethamine and mefloquine resistance emerged here [5,6].

In most of Thailand today, uncomplicated falciparum malaria is treated with artesunate-mefloquine (AM) [7]. This drug combination was first introduced in 1994 and it was adopted nationwide in 2005. This combination is also used in the surrounding countries of Cambodia and Myanmar [7].

Along the Thai-Cambodian border, AM efficacy has been declining, with 42-day parasitological cure rates of 79-83% [8-10]. AM appears more efficacious near the Thai-Myanmar border, with 42-day parasitological cure rates of >90% [9,11]. However, a recent report suggests efficacy may be starting to decrease in this area as well [12]. Since combination therapy was used in these trials, it is unclear if the increasing treatment failure is due to worsening mefloquine resistance, development of artesunate resistance, or both.

Though not an ideal approach, measuring parasite clearance time (PCT) is one way to help differentiating artesunate from mefloquine resistance in trials of AM. PCT is the time it takes for the parasitaemia to clear after anti-malarial treatment. PCT is more strongly determined by artesunate than mefloquine due to artesunate’s quick onset of action [13-17]. Therefore, a prolonged PCT would suggest increasing artesunate resistance. A recent study suggested that monitoring the presence of day-3 parasitaemia is a useful predictor of 42-day recrudescence after taking AM [18]. Based on The World Health Organization (WHO)’s working definition of artemisinin resistance, areas with 10% or greater prevalence of parasitaemia on day 3 should be further evaluated especially with a trial of artemisinin monotherapy [19].

The purpose of this study is to determine if PCT of AM varies by region and over time in Thailand and to assess the ability of the presence of parasitaemia on day 2 and day 3 to predict 28 day clinical and parasitological failure of AM. Because of loss to follow-up, day-2 parasitaemia measurement has the potential to be a more practical surveillance tool.

## Methods

### Study sites and data collection

**Figure 1:**
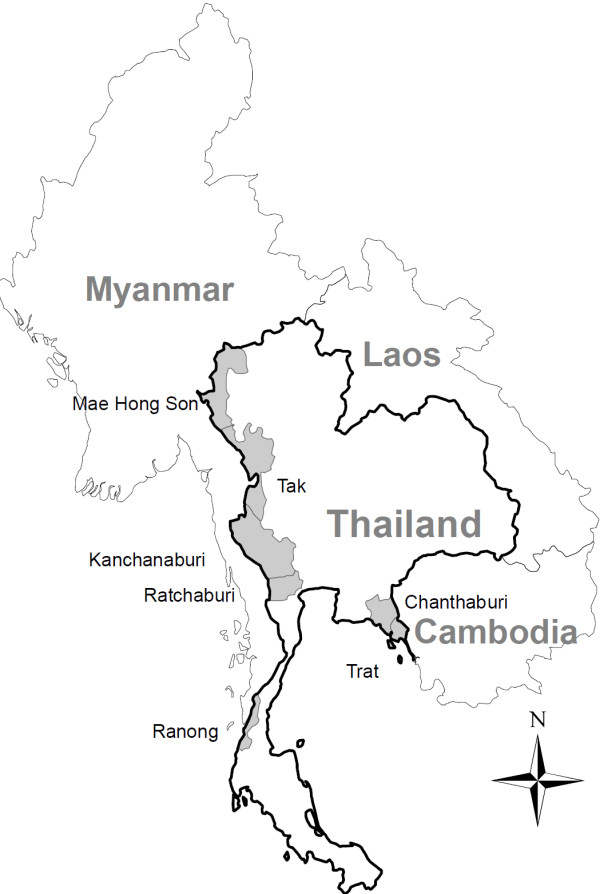
The provinces where in vivo efficacy studies took place in Thailand between 1997–2007.

Data was used from the therapeutic efficacy studies (TES) conducted by the Thai Ministry of Public Health between 1997–2007. These drug resistance monitoring sites are located in the border provinces of Thailand, where malaria is endemic (Figure 1). Of the seven sites, five were located in western Thailand (Mae Hong Son, Tak, Kanchanaburi, Ratchaburi, and Ranong) and the rest are located in eastern Thailand (Chanthaburi, Trat). Each site was not necessarily visited every year (Table 1). The WHO 1996 protocol for TES was followed until 2003, when the revised WHO protocol was adopted [20-22]. Inclusion criteria included: pure uncomplicated falciparum malaria with asexual parasite density between 500 and 120,000 parasites/μl, five years old and older, axillary temperature ≥37.5 °C (or history of fever within 24 hours), and informed consent of the patient/guardian. Pregnant women and patients with signs of severe disease were excluded.

**Table 1:** Sample sizes for in vivo efficacy studies of mefloquine 25 mg/kg + artesunate 12 mg/kg

**Region**	**Site**					**Year**				
		**1997**	**1998**	**2002**	**2003**	**2004**	**2005**	**2006**	**2007**	**Ref**
West	Mae Hong Son						103	189		
West	Tak	36		41	68	45	32	23	30	[32,33]
West	Kanchanaburi						32		38	
West	Ratchaburi						81	57		
West	Ranong				37	43	7	14	59	
East	Chanthaburi			33	44	4	6	3		[33]
East	Trat	53	37	72	44	15	20	31	30	[33]
	Total	89	37	146	193	103	281	317	157	

Briefly, at enrolment, subjects were administered mefloquine 25 mg/kg in divided doses on day 0 and artesunate 12 mg/kg divided between days 0 and 1. The maximum artesunate dose was 600 mg and for mefloquine it was 1,250 mg. This two-day regimen, as opposed to the WHO-recommended three-day regimen, was according to the standard treatment guidelines for uncomplicated falciparum malaria of the Thai National Malaria Control Programme during the study period. All drug administration was observed and repeated if there was vomiting within 30 min.

Asexual parasite density was measured using Giemsa-stained thick blood films on days 0, 2, 3, 7, 14, 21, and 28 days after enrolment. Seven additional trials extended the follow-up for 42 days. For these trials, parasite density was also measured on days 35 and 42.

Treatment outcome was defined according to the WHO protocol [20,21]. Because the trials differed in their length of follow-up (28–42 days), treatment outcome at day 28 was used so the trials could be compared. Of note, the definition of treatment outcome slightly differs between the 1996 and 2003 WHO guidelines. Therefore, the subjects from the earlier studies were reclassified in accordance to the 2003 guidelines. Briefly, the 2003 guidelines define treatment outcome as follows: Early treatment failure is the development of severe malaria on days 1, 2, or 3 in the presence of parasitaemia, day-2 parasitaemia higher than day 0, day-3 parasitaema greater or equal than 25% of the day 0 count, or the presence of parasitaemia on day 3 and with an axillary temperature greater or equal to 37.5 °C. Late treatment failure (which combines both late clinical and late parasitological failure) is the development of severe malaria after day 3, the presence of parasitaemia and fever after day 3, or the presence of parasitaemia after day 7 irrespective of temperature. Adequate clinical and parasitological response (ACPR) is the absence of parasitaemia on day 28 and not meeting the criteria for early or late treatment failure.

There was no PCR correction of the samples with positive parasitaemia and therefore re-infections and recrudescence could not be distinguished.

### Data analysis

The main questions being addressed in the analysis are: 1) does the presence of parasitaemia on day 2 and/or day 3 predict treatment failure? 2) Do the sites with higher rates of day-2 and −3 parasitaemia have higher rates of treatment failure as well? 3) How does day-2 and −3 parasitaemia vary by region and over time? Treatment failure is defined as either early or late treatment failure according to the WHO guidelines [21]. The presence of day-2 (or day-3) parasitemia is defined as the presence of peripheral parasites as detected by thick smear on day-2 (or day-3).

Chi-square, t-tests, and linear regression were used to explore the relationship between covariates and region and time. Age and parasite density were natural logarithm (ln)-transformed to meet the assumption of normality in the univariate analyses. Logistic regression was used to examine the association between the presence of day-2 and day-3 parasitaemia and region and time. Age and initial parasitaemia were included in the model a priori to control for potential confounding: both factors had the potential to vary at the different sites and over time and both have been associated with PCT (though an association between PCT and initial parasitemia is more consistently found than that between PCT with age) [17,18].

Both age and parasitaemia were coded as categorical variables with three levels while year was coded as a continuous variable. These coding decisions were made so that the variable would best reflect the relationship of the variable with the presence of day-2 and day-3 parasitaemia while taking in account the precision of the estimate. Since age was missing from all the subjects in Tak in 2006, multiple imputation was done with age to prevent this site from being excluded in the final model. Briefly, a regression model with initial parasite density, weight, sex, and region as the covariates was used to predict the age of the individuals with missing information on age.

A sensitivity analysis was done to explore the effect of outcome misclassification on the calculation of the sensitivity and specificity of the presence of day-2 parasitaemia as a marker of treatment failure. This outcome misclassification was assumed to be non-differential with regard to day-2 parasitaemia. Corrected sensitivity and specificity estimates of the presence of day-2 parasitaemia as an indicator of treatment failure were calculated using estimates of sensitivity and specificity of the 28-day uncorrected outcome as a measure of 42-day PCR-corrected outcome [23-26]. These estimates were derived from the literature. Three studies were found that met the following criteria: 1) took place in the Greater Mekong sub-region; 2) subjects received mefloquine 25 mg/kg and artesunate 12 mg/kg; and, 3) provided enough detail to derive sensitivity and specificity estimates [23,24,27].

All statistical analyses were done in Stata 11.1.

These studies were approved by the Ethical Committee for Research in Humans of the Thai Ministry of Public Health. Institutional Review Board at UNC Chapel Hill approved secondary analysis of this dataset.

## Results

### General characteristics

In total, 1,327 subjects from 31 TES at seven different sites spanning 11 years were included in this analysis (Table 1). The subjects in the east were older, had highest initial parasite density, and were more often male (Table 2). Age was missing from 61 subjects, including all the subjects at Tak in 2006. The mean age slightly decreased over time (linear regression of natural logarithm (age): β = − 0.018, t = 3.73, p < 0.001). Initial parasite density also slightly decreased over time (linear regression of natural logarithm (initial parasitaemia) and year: β = −0.026, t = 2.11, p = 0.035).

**Table 2:** General characteristics of the participants for in vivo efficacy studies by region

		**Total**	**East**	**West**	p-value^*^
Age	mean (95% CI)	31.6 (30.8, 32.3)	35.2 (33.9, 36.5)	30.0 (29.1, 30.9)	p < 0.001
	range	(5, 80)	(8, 74)	(5, 80)	
	missing	61	7	54	
Weight (kg)	mean (95% CI)	52.9 (52.3, 53.5)	55.2 (53.9, 56.4)	52.3 (51.6, 53.0)	p < 0.001
	range	(15, 92)	(18, 85)	(15, 92)	
	missing	223	164	59	
Initial parasite	geometric mean (95% CI)	17998 (16861, 19135)	20478 (18189, 22766)	16958 (15663, 18253)	p = 0.007
density	range	(520, 116960)	(560, 116000)	(520, 116960)	
	missing	0	0	0	
Sex	male (%)	1071 (81.1)	334 (85.2)	737 (79.4)	p = 0.014
	female (%)	249 (18.9)	58 (14.8)	191 (20.6)	
	missing	7	0	7	

Abbreviations: OR, odds ratio; CI, confidence interval.

^*^Results of t-tests (age, weight, and initial parasite density) and chi-square test (sex). Both age and parasite density was natural logarithm transformed.

**Figure 2:**
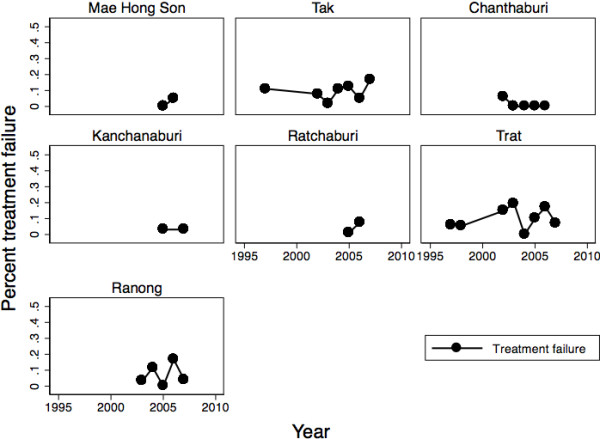
**28-day treatment failure over time in Thailand 1997–2007.** Chanthaburi and Trat are in the east, the rest of the sites are in the west.

Overall, 6.5% of the subjects experienced treatment failure by 28 days (82/1260). Sixty-seven subjects were lost to follow-up. Twenty-eight-day treatment failure varied by site (ANOVA, F = 3.85, df = 6 p < 0.001; Figure 2). Overall, treatment failure was higher in the east (34/379, 9.0%) than west (48/881, 5.5%; Chi-square = 5.40, p = 0.020). There was no change in treatment failure over time (linear regression, β= − 0.003, 95%CI: -0.008, 0.002). When controlling for age and initial parasite density, treatment failure remained higher in the east and there was still no change over time (region: OR = 1.92, 95%CI: 1.12, 3.31; year: OR = 1.01, 95%CI: 0.92, 1.11).

### Temporal and spatial patterns of day-2 and -3 parasitaemia

**Figure 3:**
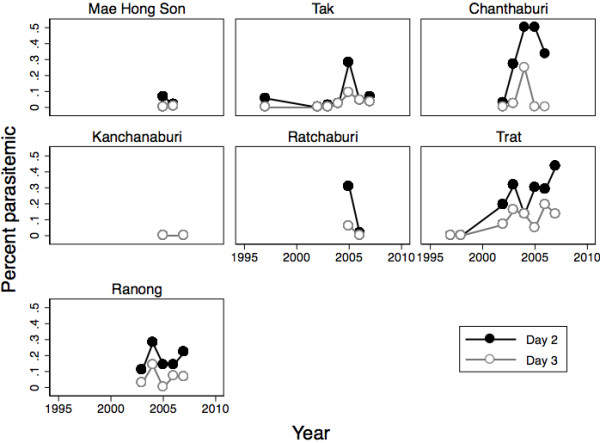
**Day-2 and day-3 parasitaemia over time in Thailand 1997–2007.** Chanthaburi and Trat are in the east, the rest of the sites are in the west.

Data on presence of day-2 and day-3 parasitaemia were available for over 99% of the subjects (day 2: 1325/1327, day 3: 1321/1327). The presence of day-2 and −3 parasitaemia varied substantially by site (ANOVA, day 2: F = 13.46, df = 6, p < 0.001; day 3: F = 6.31, df = 6, p < 0.001; Figure 3). The presence of parasitaemia on days 2 and 3 were more common in the east than the west (day 2: east: 20%, west: 9%, OR 2.47, 95% CI: 1.77, 3.45; day 3: east: 7%, west: 3%, OR = 2.79, 95%CI: 1.60, 4.87). The odds of day-2 parasitaemia increased on average 10% per year (OR = 1.10, 95% CI: 1.03, 1.19; Table 3). The increase for day 3 was similar, though the estimate was less precise (OR = 1.12, 95% CI: 0.98, 1.27; Table 4).

**Table 3:** Factors associated with the presence of day-2 parasitaemia after treatment with mefloquine and artesunate 12 mg/kg*

**Variable**	**Level**	**Day-2 parasitaemia (n)**		**Univariate**		**Multivariable**	
		**yes**	**no**	**OR**	**95% CI**	**OR**	**95% CI**
Region	East	77	315	2.47	(1.77, 3.45)	4.10	(2.75, 6.12)
	West	84	849	1.^†^		1.	
Year	1997	2	86	1.10^‡^	(1.03, 1.19)	1.27	(1.17, 1.38)
	1998	0	37				
	2002	15	130				
	2003	31	162				
	2004	17	90				
	2005	51	230				
	2006	17	300				
	2007	28	129				
Age	≤18	22	213	1.		1.	
	19-29	81	585	1.34	(0.82, 2.20)	1.33	(0.80, 2.20)
	≥30	49	314	1.51	(0.89, 2.57)	1.29	(0.73, 2.28)
Parasitaemia	<10000	56	608	1.		1.	
(parasites/μl)	10000-50000	83	466	1.93	(1.34, 2.77)	2.02	(1.39, 2.93)
	>50000	22	90	2.65	(1.54, 4.56)	2.70	(1.53, 4.74)

Abbreviations: OR, odds ratio; CI, confidence interval.

^*^Results from both univariate and multivariable logistic regression are presented. Multiple imputation was used for age in the multivariable but not the univariate analyses.

^†^reference level.

^‡^Year was coded as a continuous variable so the OR represents the comparison per one year increase in time.

**Table 4:** Factors associated with the presence of day-3 parasitaemia after treatment with mefloquine and artesunate 12 mg/kg*

**Variable**	**Level**	**Day-3 parasitaemia (n)**		**Univariate**		**Multivariable**	
		**yes**	**no**	**OR**	**95% CI**	**OR**	**95% CI**
Region	East	27	365	2.79	(1.59, 4.90)	4.69	(2.48, 8.88)
	West	24	905	1.^†^		1.	
Year	1997	0	89	1.12^‡^	(0.98, 1.27)	1.29	(1.12, 1.49)
	1998	0	37				
	2002	5	140				
	2003	9	180				
	2004	10	97				
	2005	9	272				
	2006	9	307				
	2007	9	148				
Age	≤18	4	229	1.		1.	
	19-29	31	635	2.79	(0.98, 8.00)	2.43	(0.84, 7.02)
	≥30	14	348	2.30	(0.75, 7.08)	1.60	(0.50, 5.14)
Parasitaemia	<10000	21	641	1.		1.	
(parasites/μl)	10000-50000	26	522	1.52	(0.85, 2.73)	1.53	(0.84, 2.79)
	>50000	4	107	1.14	(0.38, 3.39)	1.04	(1.34, 3.19)

Abbreviations: OR, odds ratio; CI, confidence interval.

^*^Results from both univariate and multivariable logistic regression are presented. Multiple imputation was used for age in the multivariable but not the univariate analyses.

^†^reference level.

^‡^Year was coded as a continuous variable so the OR represents the comparison per one year increase in time.

When controlling for age and initial parasitaemia, region and year remained associated with the presence of parasitaemia on day 2 (Table 3). Unfortunately, site could not be controlled for in the multivariable analysis, as the variable caused substantial collinearity with region in the logistic regression model. When the analysis was restricted to Trat (east), Tak (west), and Ranong (west), which were the sites most consistently sampled over time, the results did not substantially differ from those shown in Table 3 (in adjusted analysis of day-2 parasitemia: OR for region = 2.88, 95%CI 1.78, 4.65; OR for year = 1.35, 95%CI: 1.23, 1.49). This suggests that different sites being sampled at each year did not influence the results. These analyses were repeated for the presence of day-3 parasitaemia and the trends were similar, though the estimates were less precise (Table 4).

### Day-2 and -3 parasitaemia and treatment failure

Overall, 12% of the subjects were parasitaemic on day 2 (161/1,325) and 4% were parasitaemic on day 3 (51/1,321). Of the 51 subjects parasitaemic on day 3, 49 were also parasitaemic on day 2. Both day-2 and −3 positivity were independent predictors of 28-day treatment failure in individuals when controlling for age and initial parasitaemia (day 2: OR = 2.82, 95%CI: 1.64, 4.86; day 3: OR = 4.16, 95%CI: 1.97, 8.78). Removing subjects who experience early treatment failure decreased the magnitude of effect (day 2: OR = 2.26, 95%CI: 1.26, 4.04 day 3: OR = 2.50 95%CI: 1.01, 6.16).

Both day-2 and −3 positivity were a specific predictor for 28-day treatment failure (day 2: 1042/1177 = 88.5%, 95%CI 86.6, 90.3; day 3: 1138/1178 = 96.6%, 95%CI 95.4, 97.6). However, both had poor sensitivity (day 2: 21/81 = 25.9, 95%CI 16.8, 36.9; day 3: 10/81 = 12.4, 95%CI: 6.1, 21.5). There was no statistical significant differences between the areas under the ROC curves for day-2 and day-3 (day 2: 0.572 day 3: 0.545; chi-square 1.65, p = 0.199).

**Figure 4:**
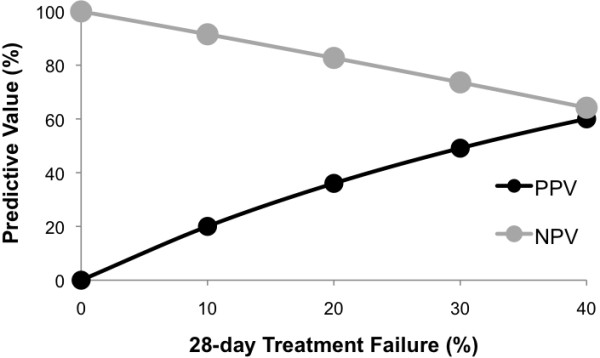
**Extrapolated positive predictive values (PPVs) and negative predictive values (NPVs).** Based on the relationship between day-2 parasitaemia and 28-day treatment failure.

The positive predictive value (PPV) was low for both day-2 and day-3 parasitaemia prevalence (day 2: 13.5, 95%CI: 8.5, 19.8; day 3: 20.0, 95%CI: 10.0, 33.7). When extrapolated for different prevalences of treatment failure, PPV remained low when the prevalence of treatment failure was less than 20% (Figure 4). Thus, both day-2 and day-3 parasitaemia predict treatment failure with similar high levels of specificity and low levels of sensitivity.

Using day 2 as a proxy for treatment failure would misclassify 60 patients that experienced treatment failure as ACPR (which is 74% of all the treatment failures). Day 3 would lead to misclassifying 71 treatment failures as ACPR (which is 87% of all treatment failures). Day 2 would misclassify 135 patients and day 3 would misclassify 40 patients as treatment failures when they were ACPR, which is 11% and 3% of the total ACPR, respectively. This suggests that day 2 and day 3 are equivalent to each other and that neither are good indicators for 28-day treatment failure.

A simple sensitivity analysis was done to explore the effect of outcome misclassification on the relationship between the presence of day-2 parasitaemia and treatment failure. When taking account for outcome misclassification (based on estimates of sensitivity and specificity for 28-day PCR-uncorrected as a proxy for 42-day PCR-corrected treatment failure derived from three separate studies), there was little difference in the sensitivity and specificity estimates of day-2 parasitaemia as an indicator for treatment failure (Table 5). Therefore, the fact that this study used the suboptimal measure of treatment failure of 28-day PCR-uncorrected treatment failure did not greatly influence the results.

**Table 5:** Sensitivity analysis of outcome misclassification^*^

	**Uncorrected**	**Alker 2007**	**Hutagalung 2005**	**Rogers 2009**
Sensitivity	25.9	22.6	15.1	15.4
Specificity	88.5	95.0	94.6	95.1
PPV	13.5	27.8	24.2	34.3
NPV	94.6	93.4	90.6	87.0

Abbreviations: PPV positive predictive value, NPV negative predictive value.

^*^The uncorrected estimates are from this study. The relationship between day-28 PCR-uncorrected treatment failure and 42-day PCR-corrected treatment failure from three different studies was used to calculate the sensitivity, specificity, PPV, and NPV of day-2 parasitaemia as an indicator of 42-day PCR-corrected treatment failure.

**Figure 5:**
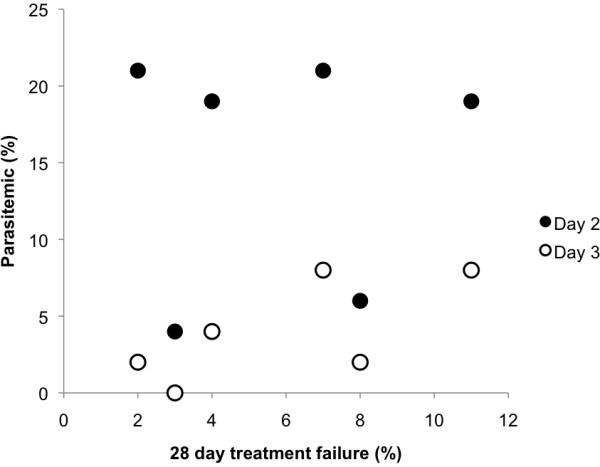
The percentage of subjects who were parasitaemic and the 28-day treatment failure.

When individual sites were compared, the percentage of subjects with either day-2 or day-3 parasitaemia showed a non-significant trend to increasing with the level of treatment failure found at that site (linear regression, day 2: β=0.76, 95%CI: -0.34, 0.49; day 3: β=0.73, 95%CI: -0.08, 1.55; Figure 5).

## Discussion

This pooled analysis from seven geographically dispersed surveillance sites in Thailand demonstrates that parasite clearance time after AM has increased between 1997 and 2007. In particular, Trat, near the Cambodian border has had a dramatic increase: the prevalence of parasitaemia on day 2 was 0% in 1997 and steadily increased to 43% in 2007. In comparison, in Tak, near the Burma border, the prevalence of delayed parasite clearance has mostly been under 10%. Of note, both initial parasite density and age changed by region and time, which may be due to differences in endemicity, environmental factors, demographics or possibly due to changing migration patterns. After controlling for these factors, day-2 parasitaemia was still increasing over time and remained higher in the east.

A previous study of a refugees’ camp in Tak in western Thailand demonstrated an increase in the prevalence of day-2 parasitaemia after AM from 3.6 to 15% from 1995–2007, which is smaller than reported here [11]. The difference is likely due to site-to-site variability and alterations in how the study drugs were given. However, this is still much lower than the prevalence of day-2 parasitaemia reported here for eastern Thailand. A small study in Pailin, Cambodia found that artesunate monotherapy had a longer parasite clearance time and a higher recrudescence rate compared in Wang Pha, in north-west Thailand [28]. However, the small sample size, the differences in baseline characteristics between the groups and technical issues in outcome determination make this study difficult to interpret [29]. Lastly, Phyo et al. recently reported an increase in the parasite clearance half-lives along the northwestern border of Thailand, with the largest increase occurring between 2008 and 2010 [30].

While it is clear that the prevalence of parasitaemia on days 2 and 3 are increasing and are higher in the east, the significance of this finding is more difficult to interpret. The presence of parasitaemia on days 2 and 3 are predictive of AM treatment failure in individuals but they have poor sensitivity and positive predictive value. However, parasitaemia on days 2 and 3 do have high negative predictive value. Therefore, the lack of parasitaemia on days 2 and 3 implies the person has a good chance of achieving adequate clinical and parasitological response to the treatment. However, the presence of parasitaemia is less informative.

In addition, the prevalence of parasitaemia on days 2 or 3 was not related to the overall treatment failure rate. While parasitaemia on day 2 is increasing over time, treatment failure rates are relatively constant. This lack of an association could be explained by delayed parasite clearance being not the best indicator of treatment failure. Another possibility is that delayed parasite clearance time is an early indicator of the emergence of artesunate resistance, which is not yet affecting the treatment failure rates.

The disconnect between day-2 and day-3 parasitaemia and treatment failure may also be due to mefloquine resistance being the main factor influencing treatment failure but having only a minor impact on parasite clearance rates. Using molecular markers of mefloquine resistance (such as *pfmdr1* genotype and copy number) may be a way to distinguish between mefloquine and artesunate decreased susceptibility and also would improve predictions of treatment failure.

When comparing day-2 and day-3 parasitaemia prevalence, there is little difference in their ability to predict AM treatment outcome. Day 2 and 3 had similar negative predictive ability but day 3 had slightly higher positive predictive power, which is consistent with previous studies [18]. ROC analysis suggests that neither are adequate surveillance tools for AM treatment failure.

The outcome in this analysis (28-day treatment failure) is suboptimal, as it is prone to misclassification as treatment failure can occur after this time period. However, a sensitivity analysis demonstrated that misclassification of this outcome has little influence of the results. In addition, the sensitivity and specificity reported here are similar to those calculated with a more stringent outcome measurement [18].

Also of note, no PCR-correction was done for the patients with recurrent parasitaemia. However, malaria incidence is generally low in most of the Mekong Sub-region, especially in Thailand. The majority of cases are occupationally exposed during a jungle trip, which typically lasts over a week. Therefore, patients who were successfully followed-up weekly in these studies were unlikely to get re-exposed to malaria in the jungles. The effect of PCR correction on the magnitude of the treatment outcomes was shown to be negligible in a similar, occupationally-exposed population of south-western Cambodia [23].

The main strength of this analysis is that this is the most extensive description of parasite clearance time following ACT reported to date, encompassing multiple sites throughout Thailand over 10 years. The main limitations are that because these data were generated from the routine drug resistance monitoring system of a national malaria control programme, metabolites for artesunate were not measured. Therefore suboptimal drug levels causing the increasing prevalence of parasites on days 2 and 3 could not be ruled out. In addition, haemoglobin E strongly influences parasite clearance time [31] and therefore geographic variations in genotype could cause the regional differences in day-2 and −3 parasitaemia seen in this study. However, haemoglobin E genotypes cannot explain the increasing day-2 and −3 parasitaemia over time in the absence of massive population migration.

The presence of parasites on day 2 is more readily measured than on day 3, as often patients return any way on day 2 for directly observed therapy. Returning again on day 3 increases the cost of in vivo studies (due to incentives) without much benefit. Both day 2 and day 3 have poor sensitivity for treatment failure in individuals, as has been seen previously [18]. The positive predictive value is low when the prevalence of treatment failure rate is less than 20%. Since the WHO recommends a treatment regimen be changed prior to it reaching that level of treatment failure, day-2 and −3 parasitaemia may not be useful on its own. In addition, this study implies that day-2 parasitaemia cannot be used as a way to detect sites with high treatment failure rates. However, day-2 parasitaemia may be a way to increase the efficiency of surveillance for treatment failures in resource-limited areas. For example, patients that remain parasitaemic on day 2 should be closely followed for treatment failure. Patients who cleared their parasites early have a much lower risk of treatment failure, and therefore could be followed more passively. Similar data evaluating day-2 and day-3 parasitaemia prevalence and its association with subsequent treatment data may be available from other sites of routine TES monitoring and could be mined to further evaluate the usefulness of and limitations of these proxy measures.

## Competing interests

The authors declare that they have no competing interests.

## Authors’ contributions

SW and WS performed site selection and implemented the trials according to WHO guidelines for Therapeutic Efficacy Study as part of the Thai National Malaria Control Programme surveillance of anti-malarial drug efficacy. AA and CW performed the data analysis. AA, SRM, CW and JRM wrote the manuscript. All authors read and approved the final manuscript.

## Disclaimer

The views expressed in this article are the views of the authors and do not necessarily reflect the official policy of USAID.
